# Abdominal complete hydatidiform mole following uterine evacuation: a case report

**DOI:** 10.3389/fonc.2026.1801023

**Published:** 2026-05-13

**Authors:** Zeqing Du, Shizhao Wang

**Affiliations:** 1 Department of Obstetrics and Gynecology, The Second Hospital of Hebei Medical University, Shijiazhuang, Hebei, China; 2 Department of Anesthesiology, The Second Hospital of Hebei Medical University, Shijiazhuang, Hebei, China

**Keywords:** abdominal pregnancy, ectopic pregnancy, gestational trophoblastic disease, human chorionic gonadotropin, hydatidiform mole

## Abstract

**Background:**

Hydatidiform mole (HM) is a form of gestational trophoblastic disease that most commonly develops within the uterine cavity. Ectopic hydatidiform mole is exceedingly rare, and abdominal implantation represents one of its rarest presentations. Due to nonspecific clinical manifestations and the lack of characteristic imaging features, abdominal HM poses significant diagnostic challenges and is frequently diagnosed only after surgical exploration.

**Case presentation:**

We report the case of a 24-year-old woman with a history of polycystic ovary syndrome who conceived following ovulation induction therapy. She was diagnosed with complete hydatidiform mole and underwent uterine evacuation. Postoperatively, serum human chorionic gonadotropin (hCG) levels failed to decline appropriately and subsequently rebounded. Imaging studies revealed no definitive intrauterine lesions, but a persistent mass was detected posterior to the uterus. Diagnostic laparoscopy identified an isolated lesion located in the rectouterine pouch and rectal peritoneum, without any communication with the uterine cavity or adnexa. The lesion was completely excised laparoscopically. Histopathological examination demonstrated hydropic swelling of chorionic villi with diffuse trophoblastic proliferation, and immunohistochemical analysis revealed absence of p57^KIP2 expression, confirming the diagnosis of abdominal complete hydatidiform mole. Following surgery, serum hCG levels rapidly declined and returned to normal. The patient recovered uneventfully and remained under close postoperative hCG surveillance, with no evidence of persistent or recurrent disease.

**Conclusions:**

Abdominal complete hydatidiform mole is a rare but important diagnostic consideration in patients with persistent or rising serum hCG levels after molar evacuation. This case highlights the limitations of imaging modalities and underscores the critical role of postoperative hCG monitoring in the early detection of ectopic molar disease.

## Introduction

Hydatidiform mole (HM) is an abnormal gestational condition characterized by disordered trophoblastic proliferation and hydropic degeneration of chorionic villi (1, 2). Based on histopathological features and genetic constitution, HM is classified into complete and partial types, with partial hydatidiform mole occasionally associated with fetal development (3, 4).The vast majority of hydatidiform moles arise within the uterine cavity, whereas ectopic hydatidiform mole is exceedingly rare (5–7).

Among ectopic locations, abdominal hydatidiform mole represents an exceptionally uncommon subtype (5).Although most ectopic pregnancies occur within the fallopian tubes, abdominal pregnancy is rare and accounts for only a small proportion of ectopic pregnancies (8, 9). Abdominal pregnancy is therefore considered a rare but potentially high-risk obstetric condition because of its substantial maternal and fetal morbidity and mortality (10, 11).

The diagnosis of ectopic hydatidiform mole is particularly challenging (12). Unlike intrauterine HM, ectopic lesions may lack characteristic ultrasonographic features, leading to delayed or missed diagnosis (5, 12). Consequently, definitive diagnosis relies largely on postoperative histopathological evaluation, supported by immunohistochemical analysis and, when available, molecular genetic testing (13–15).

Here, we report a rare case of peritoneal implantation of complete hydatidiform mole diagnosed after uterine evacuation for intrauterine complete hydatidiform mole. Because extrauterine trophoblastic lesions may raise concern for gestational trophoblastic neoplasia (GTN), careful clinicopathological correlation and postoperative serum β-hCG surveillance are essential for diagnosis, risk assessment, and timely management (16, 17). This unusual clinical course expands current understanding of the potential presentations of ectopic hydatidiform mole and highlights the diagnostic challenge of distinguishing localized implantation from overt metastatic trophoblastic disease (16, 18).

## Case presentation

A 24-year-old woman (gravida 1, para 0) with a history of polycystic ovary syndrome characterized by long and irregular menstrual cycles was referred to our hospital because of persistently elevated serum human chorionic gonadotropin (hCG) levels following uterine evacuation for hydatidiform mole. The patient had conceived after ovulation induction therapy.

Initial evaluation in early pregnancy revealed markedly elevated serum hCG levels (18,335.78 mIU/mL), which increased rapidly to 52,639 mIU/mL within one week. Transvaginal ultrasonography suggested a molar pregnancy, and the patient subsequently underwent ultrasound-guided suction curettage at a local hospital. Histopathological examination of the evacuated uterine tissue demonstrated hydropic swelling of chorionic villi with diffuse trophoblastic proliferation and absence of p57^KIP2 expression, confirming the diagnosis of complete hydatidiform mole.

**Figure 1:**
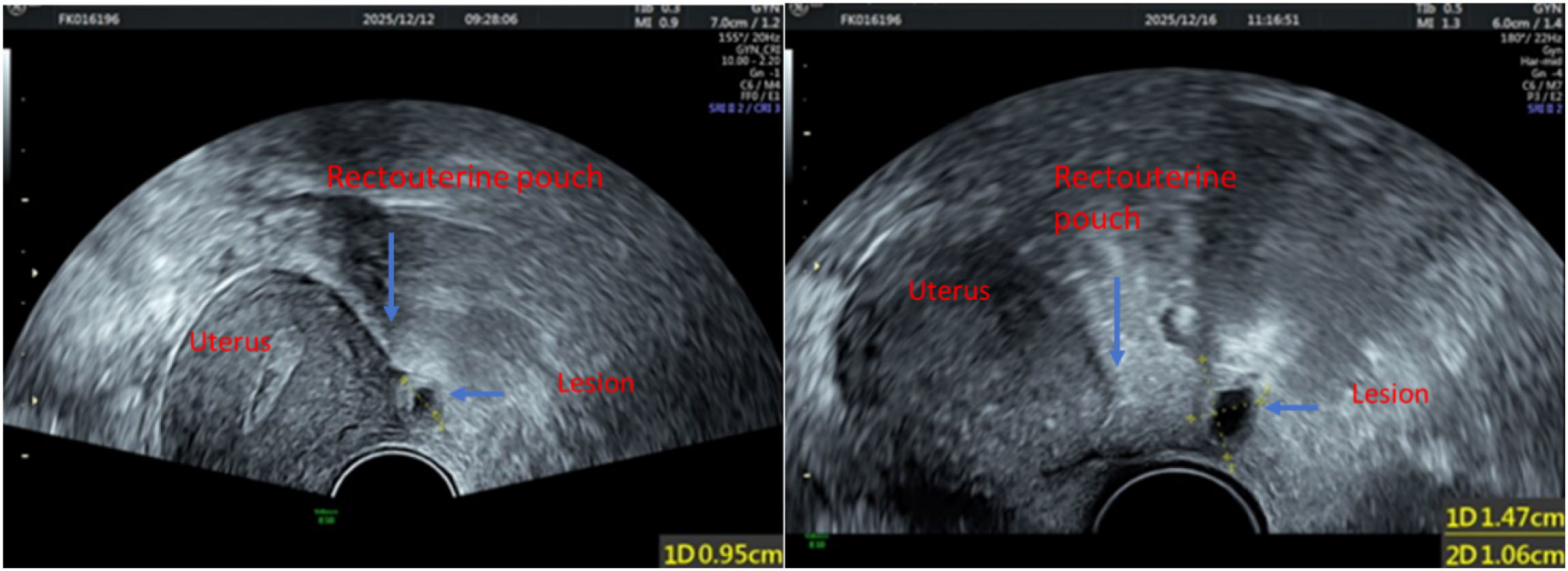
Transvaginal ultrasonography obtained at a 4-day interval (left: initial scan; right: follow-up scan), demonstrating heterogeneous endometrial echoes and a lesion located posterior to the uterus (transverse view). Key anatomical structures are labeled, including the uterus, the lesion, and the rectouterine pouch (pouch of Douglas).

**Figure 2:**
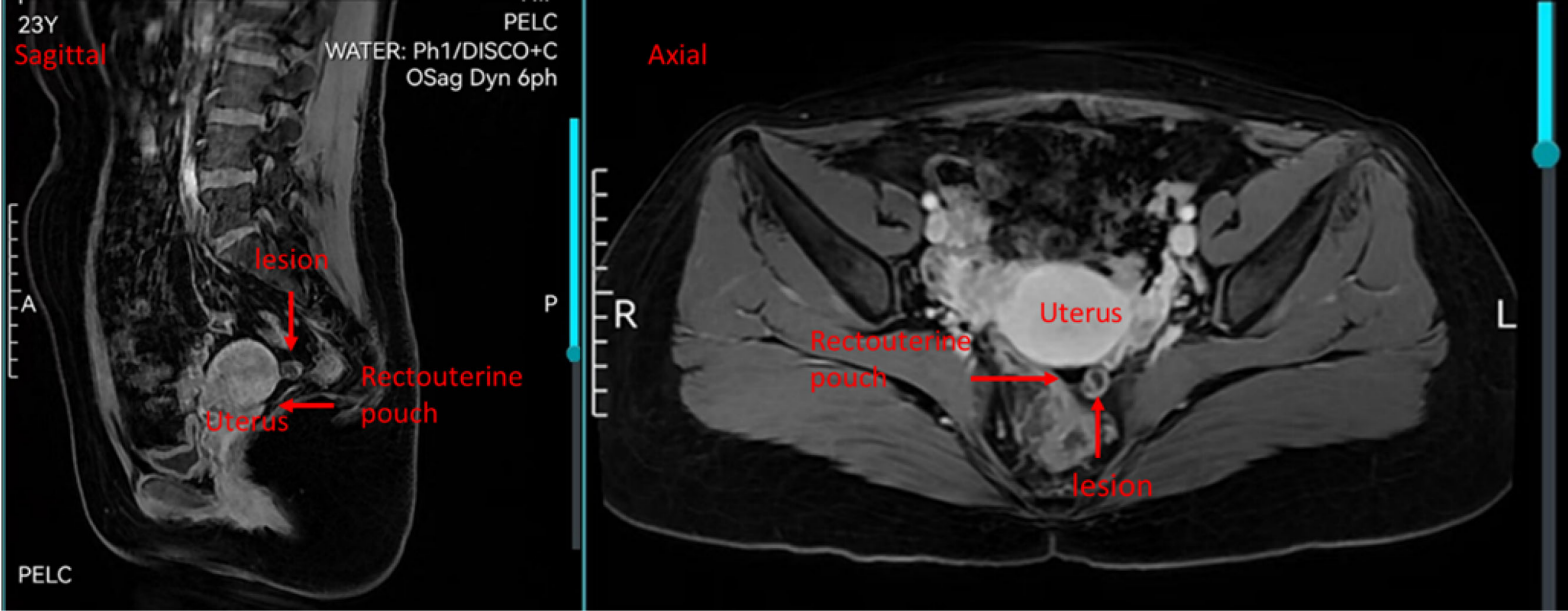
Pelvic magnetic resonance imaging (MRI) findings. Left: sagittal pelvic MRI showing a subtle lesion posterior to the uterus within the rectouterine pouch (pouch of Douglas) (arrow). Right: axial pelvic MRI demonstrating the corresponding posterior pelvic lesion (arrow). The lesion was more clearly appreciated on retrospective review of the MRI images.

**Figure 3:**
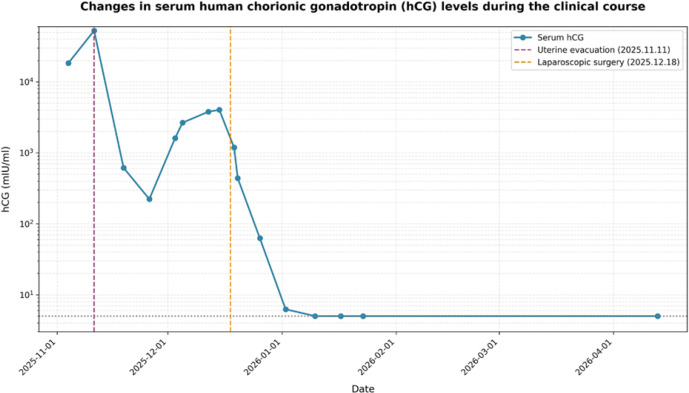
Changes in serum human chorionic gonadotropin (hCG) levels during the clinical course. The serum hCG level initially declined after uterine evacuation, subsequently rebounded before laparoscopic surgery, and then rapidly returned to the normal range after complete excision of the peritoneal lesion. The vertical dashed lines indicate the timing of uterine evacuation and laparoscopic surgery. The horizontal dotted line indicates the upper limit of the normal range.

Postoperatively, serum β-hCG levels initially declined but rebounded to 2,653.4 mIU/mL approximately three weeks later and subsequently increased further, reaching 4,037 mIU/mL before diagnostic laparoscopy. The patient reported intermittent vaginal bleeding and mild lower abdominal discomfort. Repeat transvaginal ultrasonography performed at a 4-day interval revealed heterogeneous endometrial echoes and an ill-defined mass posterior to the uterus (**Figure 1**). Whole-abdomen computed tomography showed no evidence of distant metastatic disease. Pelvic magnetic resonance imaging was also performed, and although the lesion was not clearly recognized on the initial interpretation, retrospective review demonstrated a subtle lesion posterior to the uterus, corresponding to the site later confirmed at surgery (**Figure 2**). Given the discrepancy between the biochemical findings and the initial imaging impression, persistent extrauterine trophoblastic disease was suspected, and the differential diagnosis included localized peritoneal implantation versus gestational trophoblastic neoplasia. Serial serum β-hCG levels during the clinical course are summarized in **Figure 3**.

**Figure 4:**
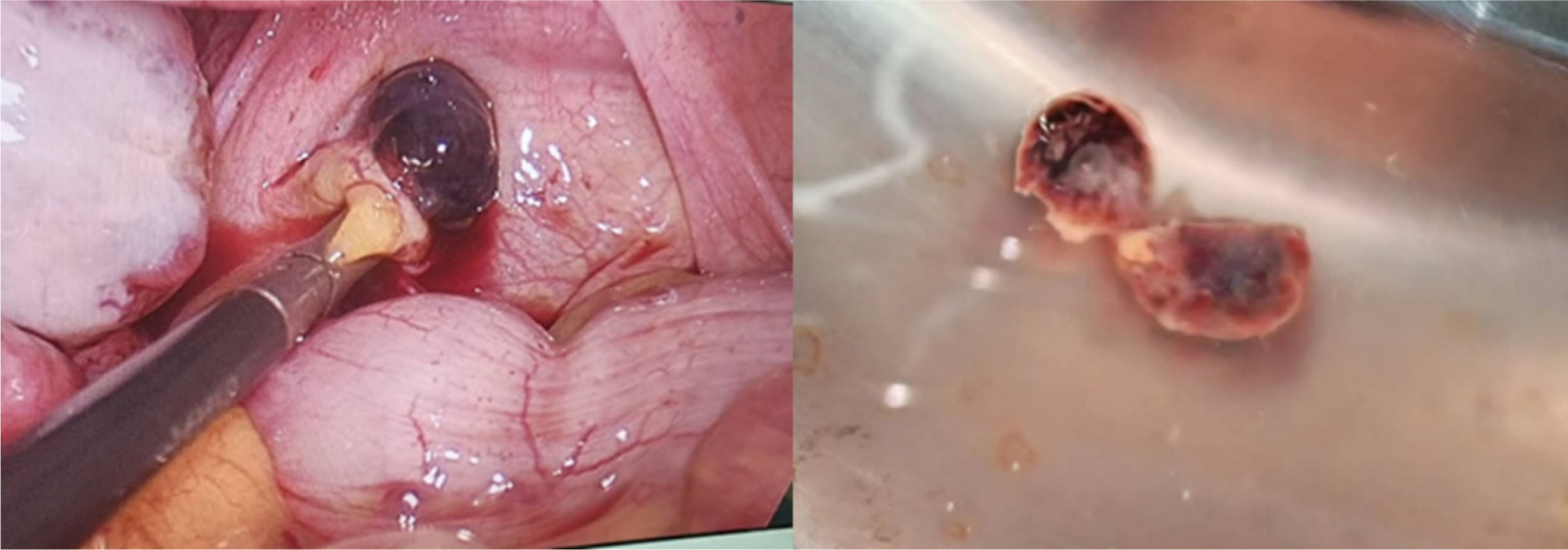
Intraoperative laparoscopic findings and gross appearance of the excised peritoneal lesion. Left: laparoscopic view showing a well-circumscribed, dark-red lesion (approximately 1 cm in diameter) located in the rectouterine pouch (pouch of Douglas), posterior to the uterus. Right: gross appearance of the lesion after laparoscopic complete excision (scale bar = 1 cm).

Diagnostic laparoscopy was subsequently performed. Intraoperatively, a small volume of hemoperitoneum was observed, and a well-circumscribed, dark red lesion measuring approximately 1 cm in diameter was identified on the left uterosacral ligament and rectal peritoneum (**Figure 4**). The uterus and bilateral adnexa appeared grossly normal, with no communication between the lesion and the uterine cavity. The lesion was completely excised laparoscopically.

**Figure 5:**
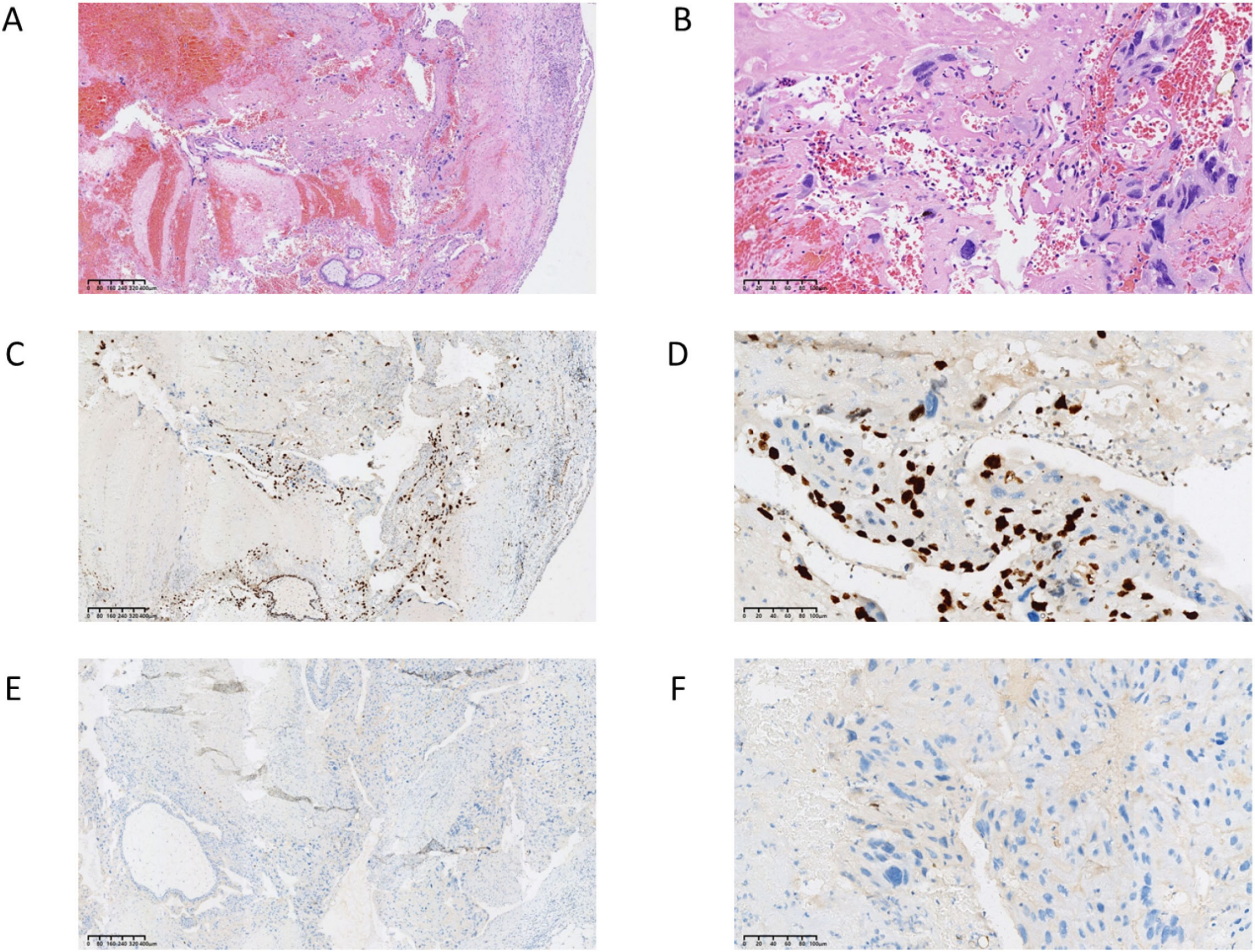
Histopathological and immunohistochemical confirmation of the peritoneal complete hydatidiform mole. **(A)** Low-magnification hematoxylin and eosin (H&E) staining (scale bar = 400 μm), demonstrating extensive hydropic degeneration of chorionic villi, hemorrhage, and overall tissue architecture of the lesion. **(B)** High-magnification H&E staining (scale bar = 100 μm), showing characteristic atypical and diffuse hyperplastic trophoblastic cells. **(C)** Low-magnification Ki-67 immunohistochemical staining (scale bar = 400 μm), highlighting widespread proliferative activity across the lesion. **(D)** High-magnification Ki-67 immunohistochemical staining (scale bar = 100 μm), showing strong diffuse nuclear positivity of trophoblasts, indicating a high proliferation index. **(E)** Low-magnification p57^KIP2 immunohistochemical staining (scale bar = 400 μm), demonstrating a complete absence of specific nuclear immunoreactivity throughout the molar tissue. **(F)** High-magnification p57^KIP2 immunohistochemical staining (scale bar = 100 μm), confirming total loss of nuclear p57^KIP2 expression in cytotrophoblasts and villous stromal cells. This immunophenotype is pathognomonic for complete hydatidiform mole.

Histopathological examination of the peritoneal lesion revealed hydropic chorionic villi with diffuse trophoblastic proliferation (**Figure 5**). Immunohistochemical staining showed absence of p57^KIP2 expression, confirming the diagnosis of peritoneal implantation of complete hydatidiform mole. Following complete laparoscopic excision, the serum β-hCG level declined rapidly and returned to the normal range. No chemotherapy was administered. The patient remained under regular serum β-hCG surveillance for 4 months postoperatively, with no evidence of persistent or recurrent disease.

## Discussion

Abdominal hydatidiform mole represents an exceptionally rare form of ectopic gestational trophoblastic disease, and its diagnosis remains challenging due to nonspecific clinical manifestations and limited sensitivity of imaging modalities (19, 20).The lesion was not clearly identified on the initial MRI interpretation because of its small size and nonspecific appearance; however, retrospective review demonstrated a subtle posterior pelvic lesion corresponding to the operative findings. This further highlights the diagnostic difficulty of extrauterine molar disease and the limitations of imaging in detecting small peritoneal trophoblastic lesions. In the present case, persistent elevation and secondary rebound of serum β-hCG levels after uterine evacuation prompted further investigation and ultimately led to the identification of a solitary extrauterine lesion in the rectouterine pouch, histopathologically confirmed as complete hydatidiform mole. This unusual presentation raised an important differential diagnostic consideration between localized peritoneal implantation and gestational trophoblastic neoplasia.

An important issue in this case was whether the extrauterine lesion should be formally classified within the conventional FIGO staging system for gestational trophoblastic neoplasia (GTN) (21, 22). According to FIGO, Stage I disease is confined to the uterus, Stage II extends outside the uterus but remains limited to genital structures, Stage III involves pulmonary metastases, and Stage IV includes all other metastatic sites (21, 23). Although the lesion in our patient was extrauterine, we believe that this case should not be mechanically assigned to the standard GTN staging framework. The lesion was solitary, located on the peritoneal surface, completely excised laparoscopically, and followed by rapid normalization of serum β-hCG without chemotherapy. These clinicopathological features were more consistent with localized peritoneal implantation of complete hydatidiform mole than with typical overt metastatic GTN. Therefore, forced classification as metastatic GTN may oversimplify the unique nature of this case and potentially be misleading. Furthermore, the absence of a need for chemotherapy, together with rapid biochemical remission after complete surgical excision and no evidence of persistent or recurrent disease during follow-up, further supported the interpretation of a localized implantation lesion rather than clinically overt GTN.

Abdominal or peritoneal hydatidiform mole represents an exceptionally rare form of ectopic gestational trophoblastic disease (24, 25). The available literature suggests that most ectopic molar pregnancies occur in the fallopian tube, whereas abdominal implantation is far less common and is therefore easily overlooked in clinical practice (26, 27). Several mechanisms have been proposed for this unusual presentation. One possibility is primary abdominal implantation of an abnormal conceptus; another, and perhaps more plausible mechanism in some post-evacuation cases, is secondary implantation or dissemination of trophoblastic tissue after uterine evacuation (24). In such cases, persistent or rising serum β-hCG after molar evacuation may be the earliest and most important clue to ongoing extrauterine trophoblastic disease (28–30). Preoperative diagnosis is often difficult because extrauterine molar lesions frequently lack specific imaging features and may not be clearly distinguished from other pelvic or peritoneal lesions on ultrasonography or magnetic resonance imaging (25, 31). Consequently, definitive diagnosis usually depends on postoperative histopathological examination, while p57 immunohistochemistry provides important support for confirming complete hydatidiform mole; when available, molecular genotyping may further improve diagnostic accuracy (32, 33). Previously reported cases indicate that management should be individualized according to lesion location, resectability, serum β-hCG dynamics, and evidence of persistent trophoblastic activity (30, 34). In selected patients with localized and resectable lesions, surgical excision may be sufficient, whereas chemotherapy is required when diagnostic criteria for gestational trophoblastic neoplasia are fulfilled (18, 30). Taken together, the published literature underscores the rarity, diagnostic complexity, and clinical importance of abdominal hydatidiform mole, and highlights the need to consider extrauterine trophoblastic disease in patients with persistent or recurrent β-hCG elevation after uterine evacuation. In the present case, the solitary peritoneal lesion, complete surgical excision, and rapid postoperative normalization of serum β-hCG without chemotherapy supported the interpretation of localized peritoneal implantation rather than typical overt metastatic GTN.

The pathogenesis of abdominal hydatidiform mole remains incompletely understood. Two principal mechanisms have been proposed: primary abdominal implantation, involving direct implantation of an abnormal fertilized ovum onto the peritoneal surface, and secondary implantation, resulting from dissemination of trophoblastic tissue following uterine evacuation or surgical manipulation (19, 35). In this case, the peritoneal lesion was anatomically separate from the uterine cavity and adnexa, and the patient had a documented history of suction evacuation for complete hydatidiform mole. These findings support secondary implantation as the more plausible mechanism. It is conceivable that microscopic trophoblastic fragments were disseminated into the peritoneal cavity during evacuation, potentially facilitated by increased intrauterine pressure or postoperative uterine contractions, as suggested in previous reports (19, 35).

Another notable aspect of this case is the patient’s history of polycystic ovary syndrome and ovulation induction therapy. Ovulation induction and assisted reproductive technologies have been reported in association with hydatidiform mole in some cases, although a causal relationship remains uncertain (36, 37).Proposed mechanisms include impaired oocyte quality, fertilization errors, and exposure to supraphysiological hormonal environments that may promote excessive trophoblastic proliferation. Although a causal relationship cannot be established based on a single case, this association highlights the importance of careful monitoring of early pregnancy outcomes following ovulation induction, particularly in patients with underlying endocrine disorders (18, 38). In clinical practice, hCG rebound is often initially attributed to residual intrauterine tissue, which may delay recognition of extrauterine disease. In the present case, imaging studies failed to identify definitive intrauterine pathology, highlighting the limitations of ultrasonography and magnetic resonance imaging in detecting small or extrauterine trophoblastic lesions (19, 20).

Immunohistochemical analysis played a pivotal role in establishing the diagnosis. The absence of p57^KIP2 expression strongly supported the diagnosis of complete hydatidiform mole, consistent with previous studies (39, 40). Although molecular genotyping represents the diagnostic gold standard, immunohistochemistry was sufficient to confirm the diagnosis in this clinical context. The absence of genomic analysis represents a limitation of this report.

## Conclusions

Abdominal complete hydatidiform mole is a rare but clinically important diagnostic consideration in patients with persistent or rising serum hCG levels after molar evacuation. This case highlights the limitations of imaging modalities and underscores the critical role of postoperative hCG surveillance. Early recognition and timely surgical intervention can lead to favorable outcomes and prevent progression to malignant gestational trophoblastic disease.

## Data Availability

The original contributions presented in the study are included in the article/supplementary material. Further inquiries can be directed to the corresponding author.
